# Assessing Quality of Life in Hepatitis C Patients: Improvements Following Direct-Acting Antiviral Therapy—A Single-Center Romanian Study

**DOI:** 10.3390/healthcare13233007

**Published:** 2025-11-21

**Authors:** Oana Koppandi, Dana Iovanescu, Bogdan Miutescu, Eyad Gadour, Oana Maria Jigau, Andreea Iulia Papoi, Calin Burciu, Eftimie Miutescu

**Affiliations:** 1 Department of Gastroenterology, Faculty of Medicine, Western University “Vasile Goldiș” of Arad, Revoluţiei Boulevard 94, 310025 Arad, Romania; koppandi.oana@uvvg.ro (O.K.);; 2 Multidisciplinary Doctoral School, Western University “Vasile Goldiș” of Arad, Revoluţiei Boulevard 94, 310025 Arad, Romania; 3 Department of Gastroenterology and Hepatology, Faculty of Medicine, “Victor Babeș” University of Medicine and Pharmacy Timișoara, Eftimie Murgu Square 2, 300041 Timișoara, Romania; 4 Advanced Regional Research Center in Gastroenterology and Hepatology, “Victor Babeș” University of Medicine and Pharmacy Timișoara, Eftimie Murgu Square 2, 300041 Timișoara, Romania; 5 Department of Gastroenterology and Hepatology, King Abdulaziz Hospital-National Guard, Ahsa 31982, Saudi Arabia; 6 Department of Internal Medicine, Faculty of Medicine, Zamzam University College, Khartoum 11113, Sudan

**Keywords:** chronic liver disease, DAAs, quality of life, SF-36v2, hepatitis C

## Abstract

**Highlights:**

**What are the main findings?**

- Direct-acting antivirals improved physical and mental quality of life in Hepatitis C patients.
- Older, female, and cirrhotic patients showed the greatest gains.

**What are the implication of the main findings?**

- Treatment benefits extend beyond a viral cure to overall well-being.
- Early therapy maximizes recovery of quality of life.

**Abstract:**

**Background/Objectives**: The Hepatitis C Virus (HCV) affects both physical health and overall well-being. The advent of direct-acting antivirals (DAAs) has greatly improved treatment outcomes. This study aimed to evaluate changes in quality of life (QoL) before and after DAA therapy. **Methods**: Ninety-seven patients with chronic HC were assessed using the SF-36v2 Health Survey, which measures eight physical and mental health domains. The questionnaire was administered at baseline (before treatment) and 12 weeks after achieving a sustained virological response (SVR). Physical Component Summary (PCS) and Mental Component Summary (MCS) scores were calculated. **Results**: Statistically significant improvements were observed across multiple domains, including Physical Functioning (mean increase: +3.99, *p* < 0.001), General Health (+5.51, *p* < 0.001), and Vitality (+4.44, *p* < 0.001). Both PCS and MCS scores improved after therapy, indicating enhanced overall well-being. Subgroup analyses suggested greater physical gains among older patients and those with cirrhosis. **Conclusions**: Successful HCV treatment with DAAs improves not only virological outcomes but also patients’ day-to-day functioning and emotional health. Routine integration of QoL assessments is essential to capture the full spectrum of benefits offered by modern antiviral therapy.

## 1. Introduction

Hepatitis C is a viral infection that can lead to chronic liver disease, cirrhosis, and even liver cancer if left untreated. Worldwide, hepatitis C remains a major public health concern, affecting roughly 50 million individuals, with nearly one million new cases emerging annually. According to the World Health Organization, in 2022 more than 240,000 deaths were attributed to hepatitis C, predominantly resulting from cirrhosis and liver cancer [1]. However, with advancements in antiviral therapy, shifting from interferon-based therapies to direct-acting antivirals (DAAs), patients can now achieve a sustained virological response (SVR), effectively curing the infection. This treatment has profound effects not only on the physical health of individuals but also on their overall quality of life (QoL) [2,3].

Globally, hepatitis C virus (HCV) remains a substantial contributor to disease burden. Between 2010 and 2019, disability-adjusted life years (DALYs) associated with acute hepatitis C declined by about 27% and those linked to HCV-related cirrhosis by 6%, reflecting progress in early disease management. However, DALYs from HCV-related liver cancer increased by over 10% during the same period, underscoring the long-term impact of chronic infection and the critical importance of early diagnosis and timely antiviral therapy [4].

In Romania, the prevalence of HCV remains relatively high compared to other European Union countries, with recent studies estimating that approximately 3% of the adult population is affected [5]. Despite broad access to DAAs through national programs, evidence on how antiviral treatment influences QoL among Romanian patients remains limited. However, a few recent studies have begun to address this topic, highlighting improvements in QoL and treatment adherence among Romanian cohorts [6,7].

Beyond hepatic disease, HCV is also linked to a wide range of extrahepatic manifestations, affecting up to two-thirds of patients in some large tertiary cohorts. These include autoimmune and lymphoproliferative disorders as well as cardiovascular, renal, metabolic and central nervous system disorders [8]. Such manifestations substantially contribute to functional impairment and psychological distress, underscoring the need to assess health-related quality of life (HRQoL) as a key outcome in HCV management.

Studies suggest that eliminating the virus can lead to improved QoL, but real-world evidence is still needed to assess how patients subjectively perceive these changes in daily life. A recent Egyptian prospective study involving 399 individuals treated with DAAs reported significant enhancements in health-related quality of life (*p* < 0.001) across all domains from baseline to 24 weeks post-treatment. The improvement was more pronounced in physical health among female participants, who were approximately 1.7 times more likely to experience physical QoL gains than males (OR = 1.69, 95% CI = 1.1–2.5 [9].

Recent studies have emphasized that treatment success in HCV is not purely biomedical but is also influenced by psychological and behavioral factors. A study conducted by Turcu-Stiolica et al. demonstrated that emotion regulation strategies significantly affect adherence to DAA therapy, and suboptimal adherence (<84%) was associated with lower SVR rates [10]. These findings highlight the central role of emotional and mental health in the overall experience and outcome of HCV therapy, supporting the importance of assessing QoL as a key dimension of treatment benefit.

A meta-analysis that included eight studies, which involved 1619 patients treated with DAAs, that evaluated QoL using the SF-36 questionnaire at baseline (T0) and again at 12 or 24 weeks post-treatment, demonstrated that DAA therapy was associated with significant and clinically meaningful improvements in most SF-36 domains, particularly in physical and mental health components. However, modest or non-significant changes were observed in certain emotional role limitation aspects [11].

Another prospective observational study conducted on 206 patients that obtained SVR and completed the EQ-5D-5L questionnaire, concluded that achieving a cure for chronic hepatitis C through DAA therapy leads to short-term gains in HRQoL, reflected by improvements in mobility, pain and discomfort, and psychological well-being indicators such as anxiety and depression. Enhancements were also observed in overall utility scores and visual analogue scale (VAS) ratings, with the greatest benefits seen among patients who initially reported lower HRQoL levels [12].

This study aims to evaluate the pre- and post-treatment QoL of patients receiving DAA therapy using the SF-36v2 Health Survey, a validated tool for assessing HRQoL. By analyzing both physical and mental health domains, this study seeks to determine the extent to which DAAs contribute to overall well-being, beyond just virological cure.

## 2. Materials and Methods

We conducted a prospective, single-center observational pre–post study at Arad County Emergency Clinical Hospital, Arad, Romania. Consecutive outpatients diagnosed with chronic hepatitis C who initiated DAA therapy between October 2020 and April 2024 were enrolled. A total of 97 patients were initially recruited, of whom 95 completed both baseline and post-treatment assessments and were included in the final analysis. Inclusion criteria comprised adult patients (≥18 years) with confirmed chronic HCV infection who completed both baseline and post-treatment SF-36v2 assessments. Exclusion criteria included decompensated liver disease, co-infection with HIV, and incomplete questionnaire data. Patients with decompensated cirrhosis were not included because such cases are managed in tertiary hepatology units. Cirrhosis was diagnosed through a combination of clinical evaluation, laboratory findings, and upper gastrointestinal endoscopy, supported by non-invasive assessments such as FibroMax^®^ and FibroScan^®^ (≥12.5 kPa) when available. The SF-36v2 Health Survey, a validated instrument that evaluates health-related quality of life across eight domains, was administered at baseline (before treatment initiation) and at 12 weeks after achieving SVR. It yields two composite scores—the Physical Component Summary (PCS) and the Mental Component Summary (MCS)—which summarize overall physical and mental health status. As this was a single-center study, findings should be interpreted with caution when extrapolating to broader populations; however, the cohort reflects real-world clinical practice within the Romanian healthcare setting.

Data were collected using paper-based SF-36v2 questionnaires administered by trained research staff during routine outpatient visits. The validated Romanian version of the SF-36v2 Health Survey, obtained under license from QualityMetric Incorporated (Lincoln, RI, USA), was used to ensure linguistic and cultural equivalence with the original instrument. Because this was an exploratory real-world study, no formal sample size calculation was performed; all eligible patients treated during the study period were consecutively included.

### Statistical Analysis

Paired *t*-tests were used to compare baseline and SVR scores for each domain and for PCS and MCS. Effect sizes were estimated using the Standardized Response Mean (SRM), with thresholds of 0.2, 0.5, and 0.8 interpreted as small, moderate, and large, respectively. Subgroup analyses were performed for gender, age (≥65 vs. <65 years), cirrhosis status, comorbidity profile and antiviral regimen. Differences between groups were examined using independent-samples *t*-tests or one-way ANOVA, as appropriate.

Responder analyses were defined as patients achieving a ≥5-point improvement in PCS, MCS, or domain scores, a threshold considered clinically meaningful. Domain-level responder rates were also calculated and reported as both absolute counts (*n*) and relative frequencies (%).

Comparisons with general population norms were performed for PCS, MCS, and domain scores. Patients were classified as below, within, or above reference values, and proportions were reported at baseline and SVR. Pearson’s correlation coefficients were used to examine associations between baseline scores and subsequent changes, as well as between PCS and MCS improvements.

All analyses were performed in Python (version 3.11) using standard scientific libraries (pandas, numpy, and matplotlib).

## 3. Results

### 3.1. Patient Demographics

The study included 95 patients with chronic HCV who received DAA therapy at Arad County Emergency Clinical Hospital, Arad, Romania, between October 2020 and April 2024. Of these, 32 (33.7%) had compensated cirrhosis and 63 (66.3%) were non-cirrhotic. Each participant completed the SF-36v2 Health Survey at two time points—baseline (pre-treatment) and 12 weeks after achieving SVR (post-treatment)—yielding a total of 190 records. Baseline demographic, clinical, and treatment characteristics are summarized in Table 1.

### 3.2. Overall QoL Changes from Baseline to SVR

Across all SF-36v2 domains, there was a statistically significant improvement in physical and mental health scores after treatment. The PCS increased from 46.66 ± 8.65 at baseline to 51.15 ± 7.29 at SVR (*p* < 0001). Similarly, the MCS improved from 50.19 ± 7.39 to 55.61 ± 3.80 (*p* < 0.001). Significant improvements were also observed in individual QoL domains. Significant improvements were also observed in individual QoL domains (Table 2). The largest gains were recorded in Role Physical (RP) (+6.03, *p* < 0.001), Mental Health (MH) (+6.36, *p* < 0.001), and General Health (GH) (+5.51, *p* < 0.001), reflecting both physical recovery and enhanced emotional well-being following viral eradication.

### 3.3. Subgroup Analysis

#### 3.3.1. Age-Related Differences in QoL Outcomes

Age was a significant determinant of baseline QoL. Patients aged ≥65 years reported lower PCS scores compared with younger patients (<65 years) (44.0 vs. 50.0), while MCS values were broadly similar.

Following SVR, both age groups showed clinically meaningful improvements. Patients aged ≥65 years achieved a mean PCS gain of +4.28 (9.7% relative increase), while younger patients improved by +4.76 (9.5%relative increase). MCS scores rose by +4.79 in older patients and +6.22 in the younger ones. Thus, while younger patients retained higher absolute scores, the magnitude of improvement was greatest among the older subgroup.

**Figure 1:**
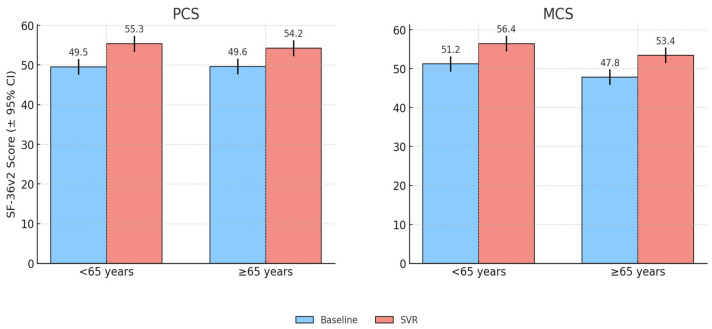
QoL scores at baseline and after SVR, stratified by age group.

Domain-level analysis revealed that RP (+6.4) and RE (+5.7) showed the largest gains in older patients, whereas GH (+8.4), MH (+8.0), and VT (+6.0) improved most among younger patients. Correlation analysis confirmed that higher age was negatively associated with baseline PCS (*r* = –0.31, *p* < 0.01), but positively associated with PCS improvement (*r* = +0.27, *p* < 0.05), indicating that older patients had more to gain from treatment. Although these correlations reached statistical significance, the coefficient values were modest (r < 0.4), indicating partial associations rather than strong predictive relationships between variables. Figure 1 illustrates the baseline and post-treatment PCS and MCS scores stratified by age group, highlighting that while younger patients consistently reported higher absolute scores, older patients achieved greater relative improvements following SVR.

#### 3.3.2. Gender-Related Differences in QoL Outcomes

Both male and female patients experienced significant improvements in PCS and MCS scores following antiviral therapy. At baseline, women had slightly lower PCS scores than men (46.1 ± 8.7 vs. 47.9 ± 8.5) although this difference was not statistically significant (*p* = 0.32). Baseline MCS values were also comparable between women and men (50.8 ± 7.1 vs. 48.8 ± 7.9, *p* = 0.24). After treatment, women improved by +4.29 points in PCS (95% CI: 2.57–6.00, *p* ≈ 6.8 × 10^−6^) and +4.45 points in MCS (95% CI: 2.50–6.40, *p* ≈ 3.2 × 10^−5^). Men showed a PCS gain of +4.95 (95% CI: 2.80–7.09, *p* ≈ 9.5 × 10^−5^) and a larger MCS improvement of +7.54 (95% CI: 5.21–9.87, *p* ≈ 6.2 × 10^−7^). Overall, both genders demonstrated clinically meaningful QoL gains, with somewhat greater improvements in mental health scores among men.

**Figure 2:**
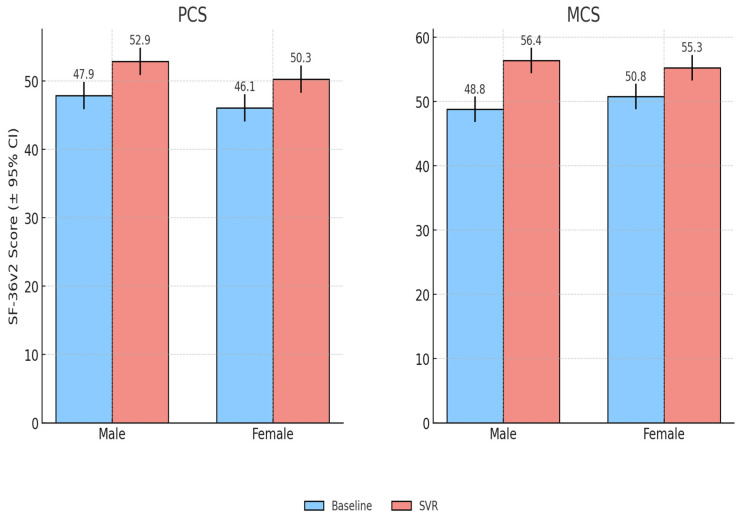
Quality of life scores at baseline and SVR, stratified by gender.

Domain-level analysis showed modest gender-specific patterns: women reported larger gains in vitality and role emotional, while men showed greater improvements in physical functioning. Importantly, the proportion of patients achieving clinically meaningful improvement (≥5 points in PCS or MCS) was similar between groups (males 72%, females 69%), suggesting that QoL benefits of viral clearance were broadly comparable across sexes (Figure 2).

#### 3.3.3. Cirrhosis vs. Non-Cirrhosis Analysis

Among the study population, 32 patients (33.7%) had compensated cirrhosis, and 63 (66.3%) were non-cirrhotic. At baseline, cirrhotic patients reported lower PCS and MCS scores than non-cirrhotic patients, indicating a greater initial impairment in both physical and mental health domains. Following SVR, both groups demonstrated significant improvements across all measured domains. Although cirrhotic patients continued to score slightly lower post-treatment, the magnitude of their improvement was sufficient to narrow the baseline gap. The largest relative recovery in cirrhotic patients was observed in the domains of GH (+7.1) and VT (+5.9), while non-cirrhotic patients showed prominent gains in RP (+5.1) and MH (+5.5).

These findings suggest that DAA therapy improves QoL irrespective of cirrhosis status, although patients with cirrhosis experience greater relative benefits due to their lower baseline scores (Table 3).

Overall, these findings indicate that although cirrhotic patients begin with more impaired QoL, they achieve improvements comparable to those of non-cirrhotic patients once SVR is reached. The narrowing of baseline disparities suggests that antiviral therapy not only restores health perceptions broadly but also reduces the QoL gap associated with cirrhosis.

### 3.4. Impact of Comorbidities on QoL

#### 3.4.1. General Effect of Comorbidities

Comorbidities were common, affecting 72.6% (*n* = 69) of patients. The most frequent were cardiovascular disease (*n* = 44, 46.3%), diabetes mellitus (*n* = 14, 14.7%), and metabolic disorders (*n* = 4, 4.2%). Other less frequent comorbidities were present in 7 patients (7.4%). Overall, comorbidity burden significantly influenced physical outcomes after SVR (F(24,66) = 1.99, *p* = 0.015), with patients carrying multiple chronic conditions showing smaller PCS gains compared with those who were otherwise healthy. In contrast, MCS improvements were broadly similar across groups, with only a non-significant trend toward lower gains in those with greater comorbidity burden (F(24,66) = 1.61, *p* = 0.065). These findings suggest that physical recovery after viral eradication may be partially limited by the presence of other chronic diseases, whereas improvements in mental and emotional well-being remain consistent across comorbidity levels.

#### 3.4.2. Cardiovascular Comorbidities

**Figure 3:**
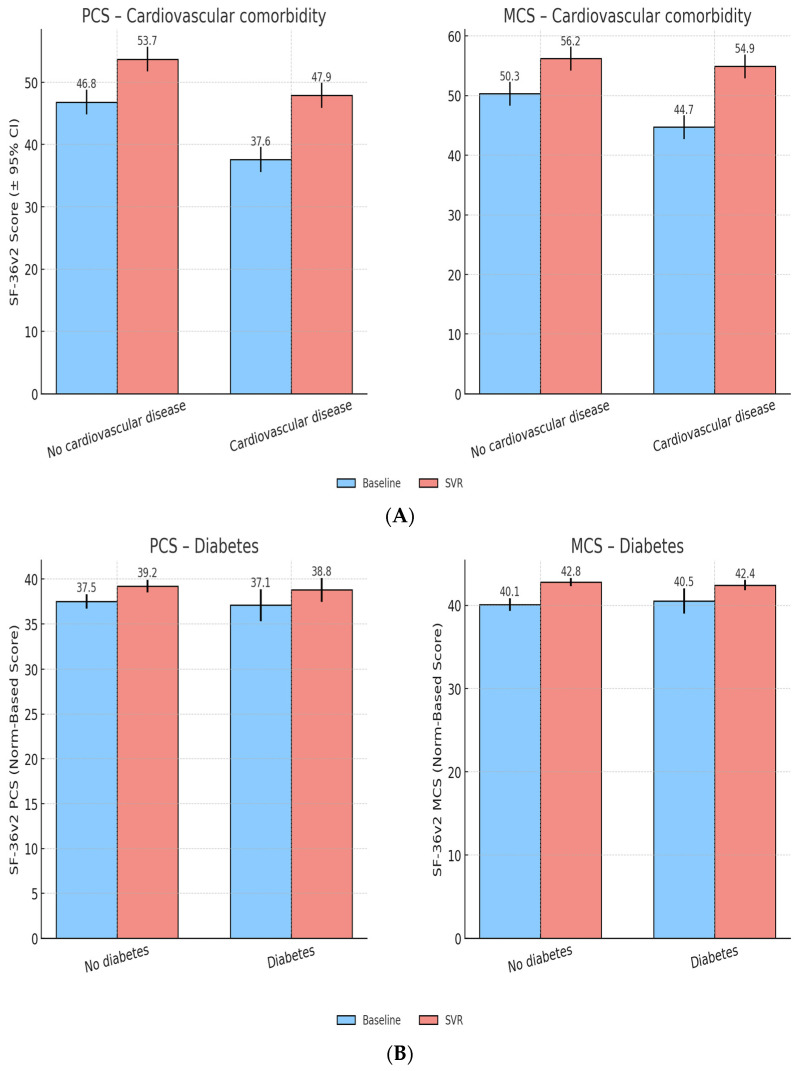
(**A**) QoL outcomes by cardiovascular comorbidity. (**B**) QoL outcomes by diabetes status.

Patients with cardiovascular comorbidities started with lower PCS (44.3 ± 8.8) and MCS (48.7 ± 7.9) scores compared to those without (PCS 48.9 ± 8.2, MCS 51.3 ± 7.1). After SVR, both groups showed improvements, though gains in PCS were smaller in the cardiovascular group (+3.8 vs. +5.2, *p* < 0.05), while MCS recovery was comparable. This suggests that cardiovascular disease attenuates physical recovery but does not prevent substantial improvements in MH (Figure 3A).

#### 3.4.3. Diabetes Mellitus

At baseline, diabetic and non-diabetic patients showed very similar QoL levels, with nearly identical PCS (37.1 vs. 37.5) and MCS scores (40.5 vs. 40.1).

Following antiviral therapy, both groups experienced modest but comparable improvements in QoL. PCS increased by +1.7 points in both diabetics and non-diabetics, while MCS improved by +2.3 and +2.7, respectively.At SVR, diabetic and non-diabetic patients reached broadly overlapping PCS and MCS scores (PCS: 38.8 vs. 39.2; MCS: 42.4 vs. 42.8), indicating that diabetes did not meaningfully limit QoL recovery in this cohort (Figure 3B).

### 3.5. Impact of Antiviral Therapy on QoL

Four DAA regimens were prescribed based on clinical criteria and national treatment guidelines: GLE/PIB (*n* = 41), LDV/SOF (*n* = 27), SOF/VEL (*n* = 16), and OMB/PAR/RIT + DAS (*n* = 11). Treatment duration was 8 weeks for GLE/PIB and 12 weeks for the other regimens. Baseline QoL scores were similar across groups, with no clinically relevant differences in PCS or MCS prior to treatment.

At SVR, PCS and MCS outcomes were broadly comparable across regimens. Patients treated with LDV/SOF tended to report slightly higher PCS scores, while those receiving SOF/VEL showed somewhat higher MCS values but these differences were not statistically significant. The GLE/PIB group, which included the largest proportion of patients, demonstrated intermediate results in both domains.

**Figure 4:**
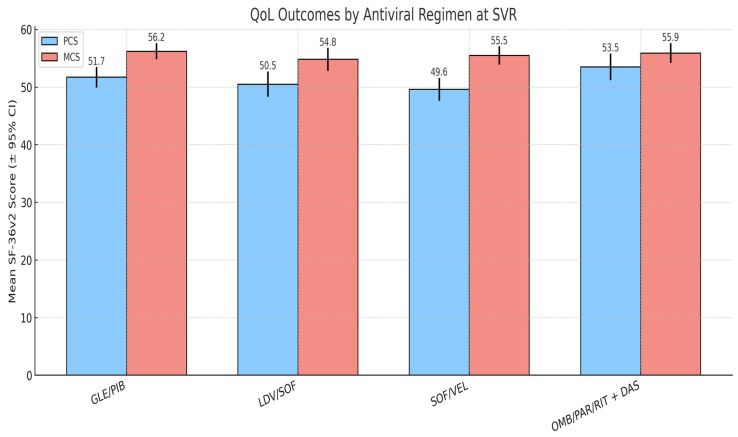
Impact of antiviral regimen QoL outcomes.

Although small numerical differences were observed, none reached statistical significance, indicating that regimen type had a minimal impact on QoL outcomes. These results suggest that the benefits of viral clearance on patient-reported well-being are consistent across commonly used DAAs, and that comorbidity burden exerts a stronger influence on post-treatment QoL than antiviral regimen choice (Figure 4).

### 3.6. Comparison with General Population Norms

When benchmarked against normative data, 58.9% of patients scored below the PCS population average at baseline, while only 4.2% were above it. After SVR, this proportion decreased to 50.5%, with nearly half of patients (49.5%) achieving PCS scores within or above the population norm. In contrast, MCS values showed a more pronounced shift: 49.5% of patients were below the normative mean at baseline, compared with only 18.9% after SVR, indicating substantial MH recovery.

The most substantial post-treatment improvements were observed in VT, GH, and PF domains, where baseline impairments were most severe. Patients who initially scored well below the norm demonstrated the largest relative gains, indicating that those with the poorest baseline status benefited the most from viral clearance.

**Figure 5:**
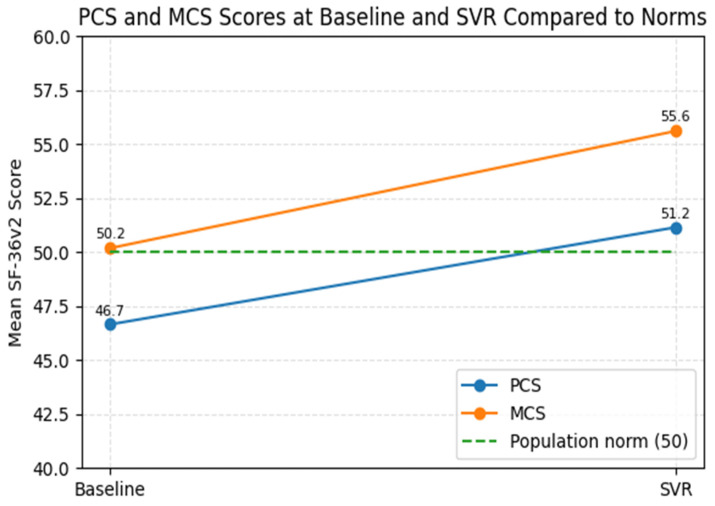
PCS and MCS scores at baseline and SVR compared with general population norms.

By SVR, mean PCS values slightly exceeded the population norm, and mean MCS scores clearly exceeded it. Together with the shift in the distribution of scores, these findings indicate a clinically meaningful improvement in MH and a partial normalization of PF (Figure 5).

### 3.7. Responder Analysis

To assess the clinical relevance of QoL improvements, responder rates were calculated using a threshold of ≥5 points improvement in summary scores. Overall, 44.2% of patients achieved a clinically meaningful gain in PCS, and 45.3% achieved a meaningful gain in MCS. These findings confirm that nearly half of the cohort experienced substantial, patient-perceived improvements in both physical and mental health after SVR.

When applying the same of ≥5 points threshold, responder rates varied across SF-36 domains. The highest proportions were observed in SF (58.9%) and MH (56.8%), followed by BP (51.6%) and RE (49.5%). PF showed the lowest responder rate (33.7%), indicating that improvements in this domain were more modest. These results suggest that while physical gains were substantial at the group level, the most consistent individual-level improvements occurred in psychosocial and pain-related domains

## 4. Discussion

This study assessed the impact of HCV treatment on patients’ QoL using the SF-36v2 questionnaire. The results demonstrate significant improvements across physical and mental domains after achieving SVR, confirming the broad benefits of viral eradication. These improvements extended across demographic groups and disease stages, although patients with comorbidities or advanced liver disease experienced more limited physical recovery. Importantly, most patients reached or exceeded general population norms, and nearly half achieved clinically meaningful gains, highlighting the substantial impact of treatment on daily functioning and well-being.

### 4.1. Comparison with Prior Romanian Studies

Our findings are in agreement with previous Romanian studies that have explored the impact of DAA therapy on QoL. Doica et al. [6] reported that higher adherence to DAA regimens was positively associated with better HRQoL outcomes, emphasizing the link between treatment compliance and patient well-being. Similarly, Pirlog et al. [7] demonstrated significant improvements in both physical and mental health dimensions following DAA treatment in Romanian cohorts with chronic hepatitis C. 

### 4.2. Thematic Synthesis of International Evidence

Internationally, numerous studies have reported parallel findings, confirming that viral eradication improves both physical and psychosocial functioning. Across cohorts from Europe, Asia, and North America, DAA therapy led to significant gains in VT, GH and emotional well-being [13,14,15,16].

Comparable results have been reported in the large ANRS CO22 HEPATHER cohort, which also confirmed improvements in HRQoL following HCV cure but identified persisting impairments among vulnerable groups. Specifically, severe liver fibrosis, unhealthy alcohol use, poverty, and female sex were associated with lower post-cure HRQoL, even after viral clearance [17].

Similar patterns were observed in the international ACTG A5360 (MINMON) trial, which demonstrated QoL improvements following DAA therapy delivered under a minimal monitoring approach [18]. Interestingly, that study reported greater improvements among patients with cirrhosis, echoing our observation that individuals with advanced liver disease, despite worse baseline scores, experienced substantial relative gains. The MINMON trial also highlighted persistent issues of anxiety and depression across countries, underscoring the importance of incorporating mental health support alongside antiviral therapy. These results, together with our data, reinforce that while DAA treatment reliably improves QoL, additional interventions may be required to optimize outcomes, particularly in vulnerable groups.

Together, these findings confirm that while HCV cure generally restores HRQoL, the extent of improvement is influenced by social determinants of health, including gender and socio-economic status. Persistent disparities related to gender, socioeconomic status, comorbidities and reduced access to post-treatment support highlight the importance of addressing broader determinants of health [17,19]. In the Romanian context, where regional and economic differences may shape access to care and recovery potential, optimizing QoL after viral eradication requires an integrated approach that combines medical, psychological, and social support. Psychological interpretation and the biopsychosocial model

HRQoL recovery after HCV cure can be understood through a biopsychosocial framework, which recognizes that physical restoration, psychological adjustment, and social reintegration are interdependent. Eradication of infection relieves physical symptoms and simultaneously reduces anxiety, stigma, and uncertainty about the future. However, patients with longer disease duration or prior psychological distress may experience slower emotional recovery [16,20,21]. Thus, integrating counseling, mental health screening, and supportive follow-up into routine care could maximize the benefits of viral clearance.

### 4.3. The Impact of Liver Disease Severity and Timing of Treatment

Across studies, cirrhosis consistently emerged as a major determinant of post-treatment quality of life. Compared to our cohort, where patients exhibited significant post-treatment improvements, cirrhotic patients demonstrated persistently lower scores, indicating that advanced liver disease may limit QoL recovery despite successful HCV treatment. In line with this, Ali et al. reported that cirrhotic patients treated with sofosbuvir-based regimens experienced marked improvements in functional, physical, and social well-being after SVR, although emotional well-being improved less consistently [15].

Together, these findings highlight that while cirrhosis remains a barrier to complete QoL restoration, meaningful recovery is still achievable, underscoring the importance of early treatment initiation before progression to advanced disease. A recent study published by Shiha et al. provides further insight into QoL recovery post-HCV treatment. Their findings reinforce the substantial QoL improvements following antiviral therapy, particularly in fatigue, vitality, and overall functional well-being. Notably, their study highlights that the degree of QoL recovery is influenced by pre-existing liver damage, with patients experiencing more significant improvements when treatment is initiated at earlier disease stages [22]. Our analysis is supported by these results, which stress the need for early detection and treatment to achieve optimal patient outcomes.

### 4.4. Long-Term Survival and Post-Cure Risks

Although achieving SVR is a major therapeutic milestone, long-term surveillance remains essential. Viral eradication does not fully eliminate the risk of metabolic, hematologic, or malignant complications—particularly hepatocellular carcinoma (HCC)—in patients with advanced fibrosis or cirrhosis [23,24,25,26,27]. Persistent liver injury and systemic inflammation may continue to affect HRQoL, even after virological cure. Therefore, comprehensive management—including metabolic control, cancer screening, and lifestyle interventions—is vital to ensure durable QoL improvement and survival beyond SVR. Limitations and future perspectives

Despite providing valuable insights, this study has certain limitations that should be considered. The relatively small sample size and single-center design may restrict the generalizability of our findings. Moreover, the absence of a control group limits the ability to establish causality. Furthermore, the follow-up period was limited to 12 weeks post-SVR, preventing evaluation of the long-term sustainability of QoL improvements.

Additionally, most of the observed correlations were of low to moderate strength, suggesting that HRQoL improvement after DAA therapy is influenced by multiple clinical and psychosocial factors rather than by any single variable. Future research should aim to address these limitations by incorporating larger, more diverse cohorts incorporating socioeconomic and psychological variables and extending follow-up periods to at least 1 year post SVR to strengthen the validity of these results.

## 5. Conclusions

This study provides important real-world evidence that DAAs substantially improve both the physical and mental dimensions of HRQoL in patients with chronic hepatitis C. These benefits extend across demographic groups and disease stages, though individuals with comorbidities or advanced liver disease may experience slower or more limited physical recovery. Most patients reached or exceeded general population norms after treatment, and nearly half achieved clinically meaningful improvements in PCS and MCS, with the highest responder rates in social functioning and mental health. These findings highlight the value of early diagnosis and timely treatment initiation and support a patient-centered approach that includes QoL assessments, mental health support, and long-term follow-ups, especially for vulnerable groups. Sustaining these benefits requires ongoing post-treatment surveillance, including metabolic and oncologic monitoring, to prevent late complications such as HCC or metabolic dysfunction.

Future efforts should prioritize long-term QoL monitoring and the integration of psychosocial support into patient care. Embedding these practices within national hepatitis programs may enhance treatment success and promote durable recovery.

## Figures and Tables

**Table 1:** Baseline demographic, clinical, and treatment characteristics study cohort (*n* = 95).

Variable	Total (*n* = 95)	Notes/Sub-Categories
Age (years)	63.1 ± 10.9	Range 33–86
Sex, *n* (%)	Female 60 (63.2%) Male 35 (36.8%)	
Cirrhosis status, *n* (%)	Compensated 32 (33.7%) Non-cirrhotic 63 (66.3%)	All decompensated cases excluded
Major comorbidities, *n* (%)	Cardiovascular disease 40 (42.1%) Diabetes mellitus 17 (17.9%) Metabolic disorders (obesity/dyslipidemia) 15 (15.8%)	
DAA regimen, *n* (%)	Glecaprevir/pibrentasvir (GLE/PIB) 41 (43.2%)	8 weeks
	Ledipasvir/sofosbuvir (LDV/SOF) 27 (28.4%)	8–12 weeks
	Sofosbuvir/velpatasvir (SOF/VEL) 16 (16.8%)	12 weeks
	Ombitasvir/paritaprevir/ritonavir + dasabuvir (OMB/PAR/RIT + DAS) 11 (11.6%)	12 weeks

**Table 2:** Improvements in individual QoL domains from baseline to SVR (*n* = 95).

Domain	Baseline Mean (SD)	SVR Mean (SD)	Mean Change	*p*-Value
Physical Functioning (PF)	45.80 (10.03)	49.78 (7.60)	+3.99	<0.001
RP	47.30 (8.27)	53.33 (5.41)	+6.03	<0.001
BP (Bodily Pain)	48.91 (8.54)	54.08 (6.71)	+5.17	<0.001
GH	46.36 (9.34)	51.88 (8.98)	+5.51	<0.001
VT (Vitality)	54.13 (7.72)	58.57 (5.59)	+4.44	<0.001
SF (Social Functioning)	45.78 (7.98)	50.37 (6.55)	+4.59	<0.001
RE (Role Emotional)	46.68 (8.18)	51.99 (5.58)	+5.31	<0.001
MH	50.68 (8.28)	57.04 (4.04)	+6.36	<0.001
PCS	46.66 (8.65)	51.15 (7.29)	+4.50	<0.001
MCS	50.19 (7.39)	55.61 (3.80)	+5.43	<0.001

**Table 3:** Comparison of SF-36v2 domain scores between patients with and without cirrhosis before and after DAA therapy.

Domain	Cirrhotic Baseline Mean (SD)	Cirrhotic SVR Mean (SD)	Mean Change	*p*-Value	Non-Cirrhotic Baseline Mean (SD)	Non-Cirrhotic SVR Mean (SD)	Mean Change	*p*-Value
BP	46.22 (8.35)	52.45 (5.58)	+6.22	0.0001	50.27 (8.38)	54.91 (7.12)	+4.64	0.0001
GH	43.74 (9.10)	50.81 (8.65)	+7.07	0.0001	47.70 (9.25)	52.42 (9.15)	+4.72	0.0001
MCS	49.07 (7.56)	55.37 (3.80)	+6.30	0.0002	50.75 (7.31)	55.73 (3.82)	+4.98	0.0001
MH	48.50 (8.92)	56.59 (3.78)	+8.09	0.0001	51.78 (7.78)	57.26 (4.17)	+5.48	0.0001
PCS	43.65 (9.07)	49.53 (7.77)	+5.88	0.0001	48.18 (8.09)	51.97 (6.95)	+3.79	0.0001
PF	42.77 (11.28)	47.73 (9.34)	+4.96	0.0001	47.33 (9.04)	50.83 (6.38)	+3.49	0.0002
RE	45.51 (8.48)	50.95 (6.06)	+5.44	0.0027	47.27 (8.03)	52.52 (5.29)	+5.25	0.0001
RP	44.80 (8.67)	52.60 (6.08)	+7.79	0.0001	48.57 (7.83)	53.70 (5.05)	+5.13	0.0001
SF	44.18 (7.93)	49.51 (6.10)	+5.33	0.0005	46.60 (7.94)	50.81 (6.78)	+4.22	0.0001
VT	52.32 (8.17)	58.26 (5.61)	+5.94	0.0001	55.05 (7.38)	58.73 (5.62)	+3.68	0.0006

## Data Availability

The dataset underlying this study contains sensitive patient information collected under conditions of confidentiality and pseudonymization, in accordance with the participants’ informed consent and the General Data Protection Regulation (EU 2016/679). Due to these ethical and legal restrictions, the full dataset cannot be made publicly available. De-identified, minimal data (aggregated SF-36v2 domain scores and summary statistics) may be made available from the corresponding author upon reasonable request, subject to institutional and ethical approval.

## Abbreviations

The following abbreviations are used in this manuscript:

HCV	Hepatitis C Virus
DAA	Direct-Acting Antiviral
QoL	Quality of Life
HRQoL	Health-Related Quality of Life
SF-36v2	Short Form (36) Health Survey, Version 2
PCS	Physical Component Summary
MCS	Mental Component Summary
SVR	Sustained Virological Response
ANOVA	Analysis of Variance
SRM	Standardized Response Mean
OR	Odds Ratio
CI	Confidence Interval
VAS	Visual Analogue Scale
DALYs	disability-adjusted life years
GLE/PIB	glecaprevir/pibrentasvir
LDV/SOF	ledipasvir/sofosbuvir
OMB/PAR/RIT + DAS	ombitasvir/paritaprevir/ritonavir + dasabuvir
SOF/VEL	sofosbuvir/velpatasvir
PF	Physical Functioning
RP	Role Physical
BP	Bodily Pain
GH	General Health
VT	Vitality
SF	Social Functioning
RE	Role Emotional
MH	Mental Health
HCC	hepatocellular carcinoma
